# ﻿Five new species of *Cranopygia* (Dermaptera, Pygidicranidae) from South China

**DOI:** 10.3897/zookeys.1262.175635

**Published:** 2025-12-10

**Authors:** Zhi-Teng Chen, Chao Jiang

**Affiliations:** 1 School of Grain Science and Technology, Jiangsu University of Science and Technology, Zhenjiang 212004, Jiangsu Province, China Jiangsu University of Science and Technology Zhenjiang China; 2 State Key Laboratory for Quality Ensurance and Sustainable Use of Dao-di Herbs, National Resource Center for Chinese Materia Medica, China Academy of Chinese Medical Sciences, Beijing 100700, China National Resource Center for Chinese Materia Medica, China Academy of Chinese Medical Sciences Beijing China

**Keywords:** Biodiversity, earwigs, genitalia, identification key, new genus record, Pygidicraninae, taxonomy

## Abstract

Five new species of the genus *Cranopygia* Burr, 1908 are described and illustrated from South China: *Cranopygia kongqueshana***sp. nov.**, *Cranopygia lisu***sp. nov.**, *Cranopygia longibifurcata***sp. nov.**, and *Cranopygia shidianensis***sp. nov.** from Yunnan Province, and *Cranopygia liuhuaishana***sp. nov.** as the first generic provincial record from Guangxi Province. These species are characterized by unique combinations of external morphological and male genital structures. Diagnostic characters, detailed illustrations, and a distribution map of these new species are provided. A provisional key to the Chinese species of *Cranopygia* is also presented based on male morphology.

## ﻿Introduction

The genus *Cranopygia* Burr, 1908 is a diverse group of earwigs belonging to the family Pygidicranidae, subfamily Pygidicraninae. Many genera and subgenera have been proposed around it, and opinions regarding their validity have long differed (Kamimura et al. 2016). *Cranopygia* is distributed throughout tropical Asia and Australasia (Hopkins et al. 2025). According to the Dermaptera Species File (DSF), the genus currently comprises 71 valid extant species (Hopkins et al. 2025). This number reflects many recent descriptions and synonymizations, yet the taxonomy of the group remains under ongoing revision (Anisyutkin 2014, 2015; Kamimura et al. 2016; Chen 2024, 2025).

Current concepts of *Cranopygia* are founded entirely on adult morphology, with emphasis on male genital structures, the configuration of the ultimate tergite and penultimate sternite, and forceps morphology. Early works recognized informal species groups within *Cranopygia*. Hincks (1959) divided the genus into five species groups based on the structure of the male forceps and genitalia, and the keys and definitions are still widely followed in the Indo–Austral and Oriental regions (Kamimura et al. 2016). Steinmann (1986) later erected several new genera to accommodate different morphological lineages, again relying heavily on male genital characters. Gorochov and Anisyutkin (1994) proposed a three-group subdivision of *Cranopygia* based on the forceps and virga structure. More recently, Kamimura et al. (2016) explicitly followed the scheme of Hincks (1959) for Oriental pygidicranines, effectively retaining the five-group classification for *Cranopygia*.

The *Cranopygia* fauna of China remains inadequately investigated. Steinmann (1986) listed seven *Cranopygia* species as occurring in China: *C. dravidia* (Burr, 1914), *C. marmoricrura* (Audinet-Serville, 1839), *C. okunii* (Shiraki, 1928), *C. proxima* Hincks, 1959, *C. sauteri* (Burr, 1912), *C. siamensis* (Dohrn, 1863), and *C. vitticollis* (Stål, 1855). Of these, *C. okunii* was regarded by Hincks (1959) as possibly related to *C. sauteri*, but its status was considered doubtful by Steinmann (1986) owing to the absence of male genitalia in the holotype. Several nomenclatural and bibliographic errors have propagated in the literature and online databases: for example, *C. dravidia* was incorrectly rendered as “*Cranopygia dravida*” in Steinmann (1986) and in the DSF, Kamimura et al. (2016) gave the year of *C. siamensis* as 1862, and the DSF gives the year of *C. marmoricrura* as 1838. Steinmann (1986) also treated *Cranopygia yunnanea* Bey-Bienko, 1959 (misspelled as “*Cranopygia yunnanea*” in the English text) as a synonym of *C. dravidia*. However, the combination of characters in *C. yunnanea*, including a virga twice as long as the external paramere, apically forked, and an unforked inner process of the external paramere (Bey-Bienko 1959; Chen and Ma 2004), contrasts with the condition in *C. dravidia* (virga slightly longer than the external paramere, apically unforked, with a bifid inner process of the external paramere; Hincks 1959). On this basis, *C. yunnanea* should be considered a valid species.

Chen and Ma (2004) later provided a different account of Chinese *Cranopygia*, listing eight species: *C. appendiculata* Hincks, 1955, *C. marmoricrura*, *C. modesta* (de Bormans, 1894), *C. proxima*, *C. siamensis*, *C. tonkinensis* Hincks, 1955, *C. vitticollis*, and *C. yunnanea*. That checklist likely reflects incomplete consultation of earlier literature and potential misidentifications. More recently, Chen (2024) described a new *Cranopygia* from Guizhou Province. Taken together, these works report 11 species in China (excluding the dubious *C. okunii*) and indicate that the true diversity of *Cranopygia* is still underestimated.

South China lies at the northern margin of the Oriental Region and forms a biogeographic bridge between the Oriental and Palearctic faunas. Several provinces in this area (e.g., Yunnan, Guizhou, Guangxi) contain tropical to subtropical habitats that are suitable for pygidicranine earwigs. Nevertheless, the *Cranopygia* fauna of China remains incompletely known and likely harbors endemic taxa (Chen and Ma 2004). In this study, we describe five new *Cranopygia* species from South China, thereby expanding the documented distribution of the genus and emphasizing the importance of this region for elucidating the biodiversity of Dermaptera.

## ﻿Material and methods

**Figure 1.**
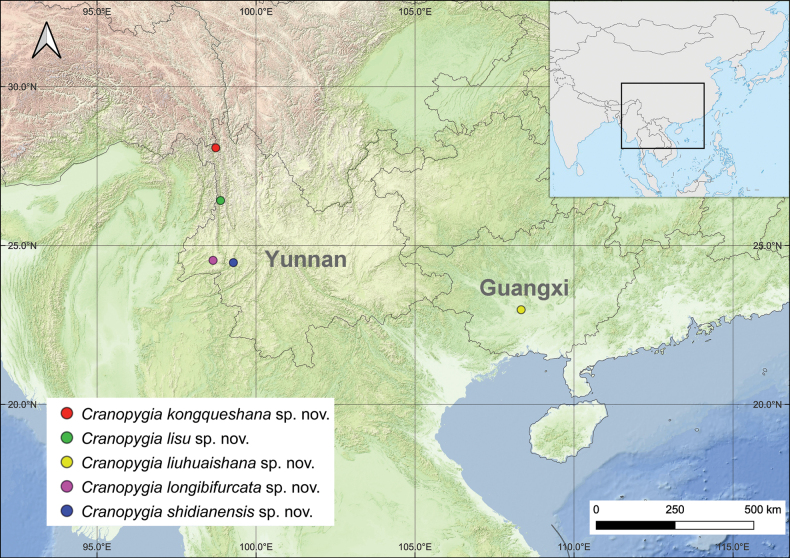
Distribution of the new *Cranopygia* species in this study.

The specimens were hand-collected from Guangxi and Yunnan provinces (Fig. 1) and preserved in 75% ethanol. The type specimens are deposited in the Insect Collection of Jiangsu University of Science and Technology (**ICJUST**), Jiangsu Province, China. Morphological examinations and identifications were conducted using an SDPTOP SZM45 stereomicroscope (Sunny Optical Technology (Group) Co., Ltd, Zhejiang, China). Images were captured as 8688 × 5792 JPG files by a Canon EOS 5DSR camera (Canon, Tokyo, Japan) with a Canon MP-E 2.8/65 mm macro lens (Canon, Tokyo, Japan). Figure plates were uncompressed and optimized in Adobe Photoshop 2025 (Adobe Inc., San Jose, CA, USA). The distribution map of the new species was produced using QGIS v. 3.40.11-Bratislava (https://www.qgis.org). Body length was measured from the anterior margin of the head to the posterior tip of the forceps. The forceps was measured from the visible lateral base to the posterior apex. The morphological terminology follows Steinmann (1986), and the species-group classification follows Hincks (1959). The Chinese translations of the names are provided in Chinese characters, followed by the relative pinyin in square brackets.

## ﻿Results

### ﻿ Cranopygia kongqueshana sp. nov.

Taxon classificationAnimaliaDermapteraPygidicranidae

94B1224F-0D72-5637-9719-05E9719EB958

https://zoobank.org/3BF38E64-4B7E-4CF1-B225-FD5AA7D59E06

Figs 2, 3

#### Specimens examined.

***Holotype***: China • ♂; Yunnan Province, Nujiang Lisu Autonomous Prefecture, Dimaluo Village, Kongque Mountain; 28.0732°N, 98.7320°E; 2500 m; 18.vii.2025. ***Paratype***: China • ♂; same data as holotype. No additional non-type material examined.

#### Differential diagnosis.

The new species belongs to the *siamensis*-group based on the genital lobe being one-fourth of the entire genitalia length, the external paramere bearing a distinct outer process, and the virga being long, strongly curved, basally sclerotized, without lateral flanges, and unforked apically (Hincks 1959). Within this group, it most closely resembles *Cranopygia guizhouensis* Chen, 2024 from Guizhou Province in the shape of the forceps and genitalia (Chen 2024). It differs from *C. guizhouensis* by the absence of broad yellow patches on the median pronotum and lateral tegmina, the genitalia six times as wide as the basal stem of the external paramere (vs. four times), the external paramere three times as long as the width of basal stem (vs. twice), the outer process twice as wide as long (vs. as long as wide), the inner process narrowly bifid (vs. widely bifid), the virga extending beyond the apex of the genital lobe when not extruded (vs. concealed within the genital lobe), and the basal vesicle near as long as the external paramere (vs. two-thirds as long).

#### Description.

**Figure 2.**
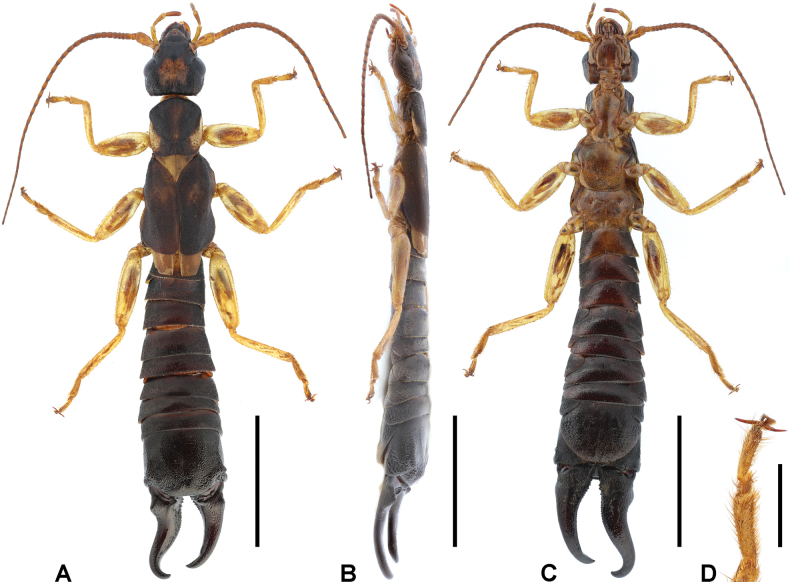
*Cranopygia kongqueshana* sp. nov., male holotype. **A.** Habitus, dorsal view; **B.** Habitus, lateral view; **C.** Habitus, ventral view; **D.** Tarsomeres of left hind leg, dorsal view. Scale bars: 5 mm (**A–C**); 1 mm (**D**).

**Male. *General appearance*.** Large-sized, whole body mostly setose (Fig. 2A–C). Body length (from anterior of head to posterior of forceps) 42.5 mm. Forceps asymmetrical; left branch length (from visible lateral base to posterior end) 7.2 mm; right branch length 7.1 mm.

***Head*.** Head longer than broad; mostly dark, medially with one six-lobed pale area. Frontal and coronal sutures obscure. Eyes not prominent, about as long as genae. Antennae mostly brown, with at least 33 segments; first antennal joint slightly shorter than distance between antennal bases. Mouthparts pale brown to dark brown.

***Thorax*.** Pronotum longer than wide; anterior and lateral margins rounded; posteromedial margin distinctly emarginate, forming two posterolateral lobes. Median longitudinal furrow distinct. Surface mostly dark, with pale median and lateral areas. Tegmina well developed, near twice as long as pronotum; mostly dark brown, with a small pale area on anterior half of dorsal surface. Scales of hindwings pale; as long as wide; posterior margins weakly rounded. Legs slender, mostly pale brown, with brown spots on dorsal surfaces of all femora; second tarsomere near as wide as third (Fig. 2D).

***Abdomen*.** Abdomen dark brown, gradually expanded to last tergite. Ultimate tergite broad, subquadrate; posteromedial extension rounded, with truncate posterior margin; weakly punctured; nearly glabrous; median longitudinal sulcus present. Forceps dark brown, subcontiguous, asymmetrical; bases strongly trigonal, with both inner-dorsal ridge and outer dorsal tooth; conical apically; inner margin crenulate nearly throughout except apex. Penultimate sternite rounded, posterior margin weakly emarginate.

**Figure 3.**
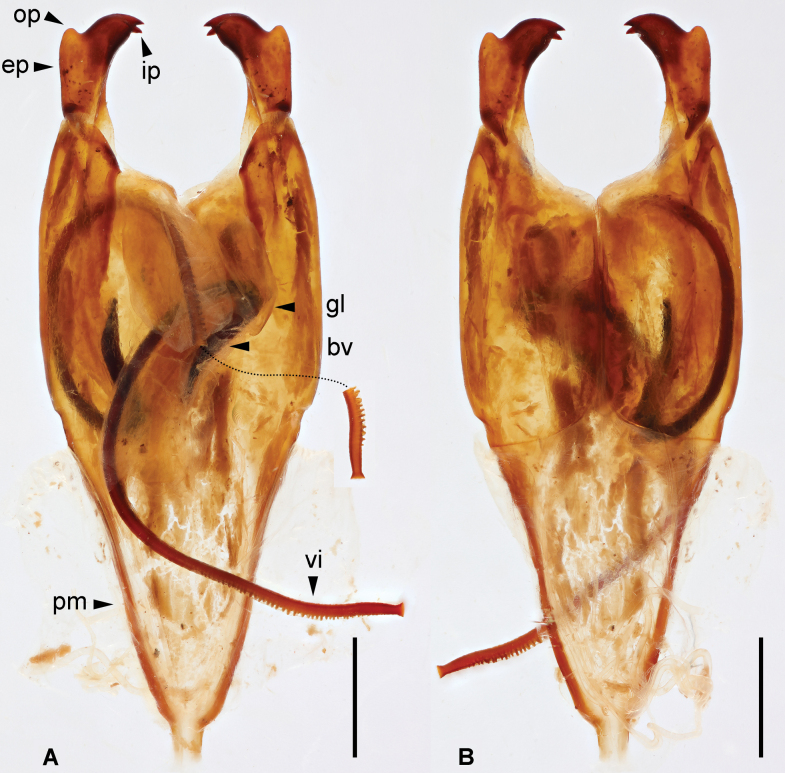
*Cranopygia kongqueshana* sp. nov., male genitalia. **A.** Dorsal view; **B.** Ventral view. Dashed line indicates the belonging of a broken virga and its apex, rather than a missing part. Abbreviations: bv – basal vesicle; ep – external paramere; gl – genital lobe; ip – inner process; op – outer process; pm – paramere; vi – virga. Scale bars: 1 mm.

***Genitalia*.** Genitalia very broad, brown (Fig. 3A, B). Paramere subtriangular basally. Genital lobes well developed; virga within genital lobe slender, apical one-third with denticulate outer margin until before apex, apex widened and truncate; basal vesicle sclerotized, widened toward apex. External paramere stout, constricted basally, widened apically; incision of anterior margin rounded, shallow, wide; inner process stout, bifid apically, apical incision subtriangular; outer process short, obtuse.

#### Etymology.

The new species is named after Kongque Mountain, the type locality.

#### Distribution.

The species is currently known only from Yunnan Province, China.

### ﻿ Cranopygia lisu sp. nov.

Taxon classificationAnimaliaDermapteraPygidicranidae

AE2D3541-6475-588D-917F-43F794BC271F

https://zoobank.org/F505A20B-E202-4BC4-86E7-8B13060A7189

Figs 4, 5

#### Specimen examined.

***Holotype***: China • ♂; Yunnan Province, Nujiang Lisu Autonomous Prefecture, Fugong County, Nujiang Grand Canyon National Park; 26.4143°N, 98.8839°E; 1800 m; 24.viii.2025; Hu Xiao, leg. No additional non-type material examined.

#### Differential diagnosis.

The new species belongs to the *siamensis*-group based on the male genital structure (Hincks 1959). It is most similar to *C. kongqueshana* sp. nov. in general habitus and genital morphology but differs by the pronotum being wider than long (vs. longer than wide), posteromedial margin weakly emarginate (vs. distinctly emarginate), inner process of the external paramere with rounded apical incision (vs. subtriangular), and virga (when in situ) outside of genital lobe near as long as genital lobe (vs. half as long).

#### Description.

**Figure 4.**
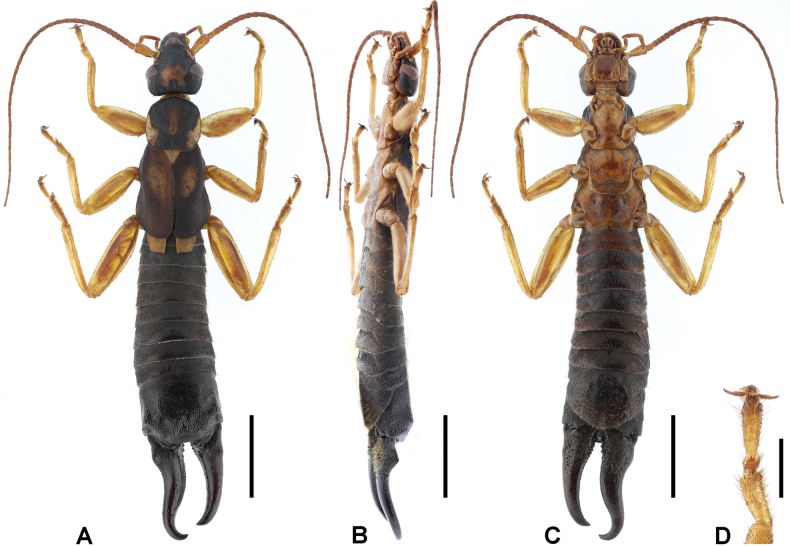
*Cranopygia lisu* sp. nov., male holotype. **A.** Habitus, dorsal view; **B.** Habitus, lateral view; **C.** Habitus, ventral view; **D.** Tarsomeres of left foreleg, dorsal view. Scale bars: 5 mm (**A–C**); 1 mm (**D**).

**Male. *General appearance*.** Large-sized, whole body mostly setose (Fig. 4A–C). Body length 31 mm. Forceps asymmetrical; left branch length 6.7 mm; right branch length 6.2 mm.

***Head*.** Head longer than broad; mostly dark, medially with one M-like pale area. Frontal sutures invisible, coronal suture obscure. Eyes not prominent, about as long as genae. Antennae mostly brown, with at least 33 segments; first antennal joint slightly shorter than distance between antennal bases. Mouthparts brown to dark brown.

***Thorax*.** Pronotum slightly wider than long; anterior and lateral margins rounded; posteromedial margin weakly emarginate. Median longitudinal furrow distinct. Surface mostly dark, with pale median and lateral areas. Visible part of mesonotum pale. Tegmina well developed, near twice as long as pronotum; mostly dark brown, with a big pale area on anterior half of dorsal surface. Scales of hindwings mostly pale, posterolateral margins dark; wider than long; posterior margins weakly rounded. Legs slender, mostly pale brown, with brown spots on dorsal surfaces of all femora; second tarsomere narrower than third (Fig. 4D).

***Abdomen*.** Abdomen dark brown, gradually expanded to last tergite. Ultimate tergite broad, subquadrate; posteromedial extension rounded, with truncate posterior margin; weakly punctured; nearly glabrous; median longitudinal sulcus present. Forceps dark brown, subcontiguous, asymmetrical; bases strongly trigonal, with both inner-dorsal ridge and outer dorsal tooth; conical apically; inner margin crenulate mostly at basal two-thirds. Penultimate sternite rounded, posterior margin weakly emarginate.

**Figure 5.**
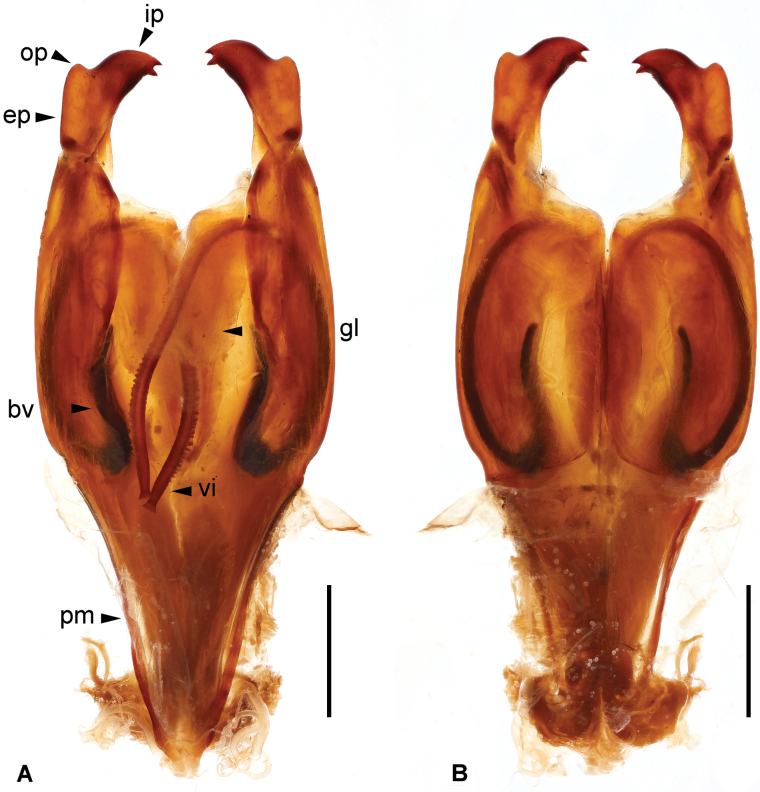
*Cranopygia lisu* sp. nov., male genitalia. **A.** Dorsal view; **B.** Ventral view. Abbreviations: bv – basal vesicle; ep – external paramere; gl – genital lobe; ip – inner process; op – outer process; pm – paramere; vi – virga. Scale bars: 1 mm.

***Genitalia*.** Genitalia very broad, dark brown (Fig. 5A, B). Paramere subtriangular basally. Genital lobes well developed; virga within genital lobe slender, apical one-third with denticulate outer margin until before apex, apex widened and truncate; basal vesicle sclerotized, widened toward apex. External paramere stout, constricted basally, widened apically; incision of anterior margin rounded, shallow, wide; inner process stout, bifid apically, apical incision rounded; outer process short, obtuse.

#### Etymology.

The species is dedicated to the Lisu nationality.

#### Distribution.

The species is currently known only from Yunnan Province, China.

### ﻿ Cranopygia liuhuaishana sp. nov.

Taxon classificationAnimaliaDermapteraPygidicranidae

DDF66745-6DDB-59AD-AF10-D1C6387277F9

https://zoobank.org/BCD6D99B-1005-4092-939C-B60365155901

Figs 6, 7

#### Specimen examined.

***Holotype***: China • ♂; Guangxi Province, Nanning City, Wuming District, Liuhuai Mountain; 22.9774°N, 108.3370°E; 880 m; 20.v.2024. No additional non-type material examined.

#### Differential diagnosis.

This species belongs to the *picta*-group, characterized by a mucronate external paramere with a poorly developed outer process and an extremely long virga that is basally sclerotized, without lateral flanges, and unforked apically (Hincks 1959). It resembles *Cranopygia manipurensis* Srivastava, 1975 in having tegmina shorter than the pronotum, a deeply notched penultimate sternite, and a virga with a medial knob (Srivastava 1975), but differs by its genital lobes and virga being about twice as long. It is also similar to *Cranopygia corymbifera* Anisyutkin, 1997 (Anisyutkin 1997), but can be distinguished by its tegmina twice as long as wide (vs. three times) and by the virga near four times as long as the genital lobe (vs. near twice).

#### Description.

**Figure 6.**
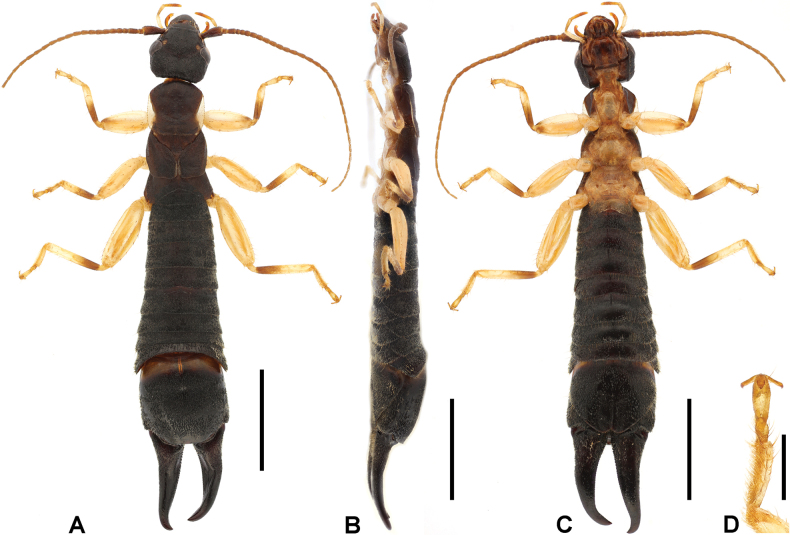
*Cranopygia liuhuaishana* sp. nov., male holotype. **A.** Habitus, dorsal view; **B.** Habitus, lateral view; **C.** Habitus, ventral view; **D.** Tarsomeres of right hind leg, dorsal view. Scale bars: 5 mm (**A–C**); 1 mm (**D**).

**Male. *General appearance*.** Median-sized, whole body mostly setose (Fig. 6A–C). Body length 26 mm. Forceps asymmetrical in shape, with same length 5.1 mm.

***Head*.** Head longer than broad; mostly dark, with small pale spot near antero-inner margin of each eye. Frontal and coronal sutures obscure. Eyes not prominent, about as long as genae. Antennae brown, with at least 31 segments; first antennal joint shorter than distance between antennal bases. Mouthparts pale brown to dark brown.

***Thorax*.** Pronotum slightly wider than long; anterior and lateral margins rounded; posteromedial margin weakly emarginate. Median longitudinal furrow distinct. Surface mostly dark brown, with pale lateral areas. Visible part of mesonotum dark brown. Tegmina strongly reduced, slightly shorter than pronotum; dark brown. Scales of hindwings pale absent. Legs slender, mostly pale, with basal half of tibia brown; second tarsomere narrower than third (Fig. 6D).

***Abdomen*.** Abdomen dark, gradually expanded to last tergite. Ultimate tergite broad, subquadrate; posteromedial extension rounded, with truncate posterior margin; weakly punctured; mostly hairy; median longitudinal sulcus obscure. Forceps dark brown, subcontiguous; asymmetrical, right branch more curved than left branch; bases strongly trigonal, with inner-dorsal ridge; conical apically; inner margin crenulate nearly throughout except apex. Penultimate sternite rounded, posterior margin deeply notched.

**Figure 7.**
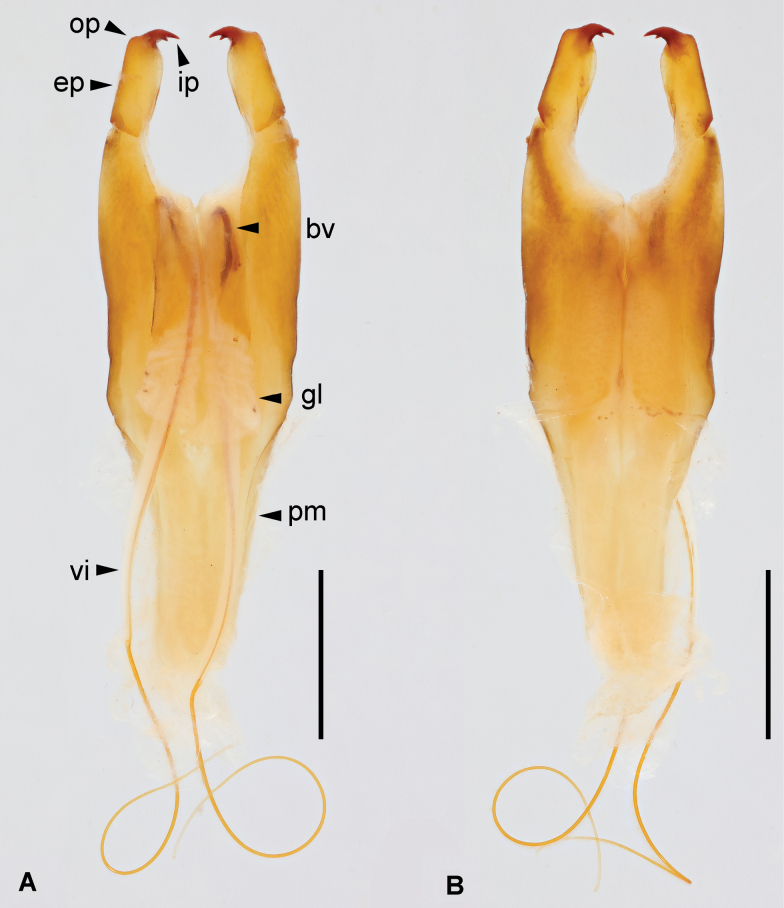
*Cranopygia liuhuaishana* sp. nov., male genitalia. **A.** Dorsal view; **B.** Ventral view. Abbreviations: bv – basal vesicle; ep – external paramere; gl – genital lobe; ip – inner process; op – outer process; pm – paramere; vi – virga. Scale bars: 1 mm.

***Genitalia*.** Genitalia slender, pale brown (Fig. 7A, B). Paramere subtriangular basally. Genital lobes well developed; virga within genital lobe thin, extremely long, with a distinct nodule near midpoint, apex widened and truncate; basal vesicle sclerotized, thicker than virga. External paramere slender, inner margin convex, outer margin straight, with mostly consistent width; inner process pointed, with a small tooth near midpoint of its inner margin; outer process weakly developed, rounded.

#### Etymology.

The new species is named after Liuhuai Mountain, the type locality.

#### Distribution.

The species is currently known only from Guangxi Province, China.

### ﻿ Cranopygia longibifurcata sp. nov.

Taxon classificationAnimaliaDermapteraPygidicranidae

2D16EB82-22D7-5DC3-BD80-36D5E0036787

https://zoobank.org/7E5446B4-9EB2-4127-8393-3444D7D8ACD8

Figs 8, 9

#### Specimen examined.

***Holotype***: China • ♂; Yunnan Province, Dehong Dai and Jingpo Autonomous Prefecture, Mang City, Kongque Valley Forest Park; 24.5313°N, 98.6468°E; 1350 m; 6.vii.2025. No additional non-type material examined.

#### Differential diagnosis.

The new species belongs to a previously undefined *appendiculata*-group, which includes *Cranopygia appendiculata* Hincks, 1955, *Cranopygia bifurcata* Srivastava, 1979, and *C. yunnanea*, characterized by symmetrical forceps, external paramere with inner and outer processes of similar size, and virga long, basally sclerotized, without lateral flanges, and apically forked. It closely resembles *C. bifurcata* in male habitus and genitalia, but differs in the penultimate sternite with convex lateral margins (vs. emarginate) and apical branches of the virga about 1.1 times as long as the external paramere (vs. about two-thirds its length) (Srivastava 1979). In addition, the new species differs from *C. appendiculata* in having apical branches of the virga of similar length (vs. one branch more than twice as long as the other), and from *C. yunnanea* in possessing apical branches of the virga slightly longer than the external paramere (vs. approximately one-third its length) and external paramere three times as long as wide (vs. twice) (Hincks 1955; Bey-Bienko 1959; Chen and Ma 2004).

#### Description.

**Figure 8.**
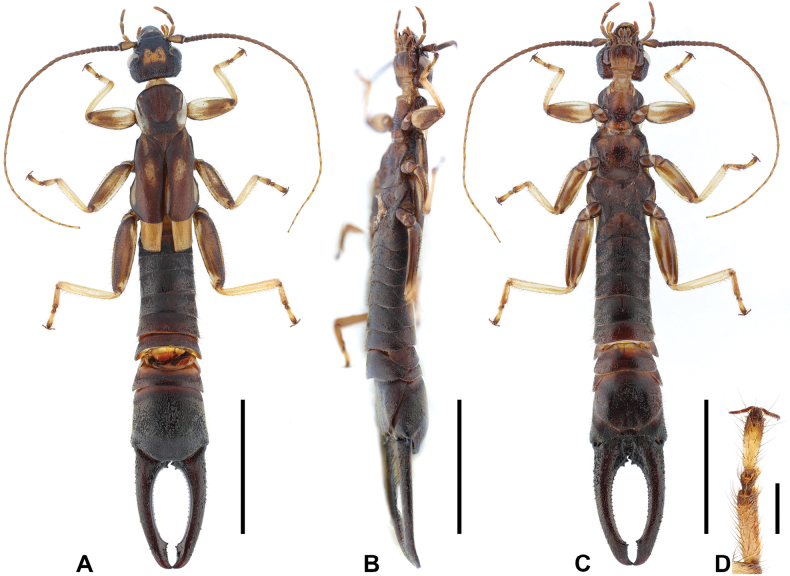
*Cranopygia longibifurcata* sp. nov., male holotype. **A.** Habitus, dorsal view; **B.** Habitus, ventrolateral view; **C.** Habitus, ventral view; **D.** Tarsomeres of left hind leg, dorsal view. Scale bars: 10 mm (**A–C**); 1 mm (**D**).

**Male. *General appearance*.** Large-sized, whole body mostly setose (Fig. 8A–C). Body length 40.5 mm. Forceps symmetrical, length 9.4 mm.

***Head*.** Head longer than broad; mostly dark, medially with one bilobed pale area. Frontal and coronal sutures distinct. Eyes not prominent, about as long as genae. Antennae mostly brown, with at least 39 segments; first antennal joint shorter than distance between antennal bases. Mouthparts pale brown to dark brown.

***Pronotum*.** Pronotum about as long as wide; anterior and lateral margins rounded; posteromedial margin emarginate. Median longitudinal furrow distinct. Surface mostly dark, with pale lateral areas.

***Thorax*.** Tegmina well developed, near twice as long as pronotum; mostly dark brown, with a continuous pale stripe on lateral surface and an interrupted pale stripe on anterior half of dorsal surface. Scales of hindwings pale; longer than wide; posterior margins weakly rounded. Legs slender, mostly pale, with brown stripes on femora; second tarsomere near as wide as third (Fig. 8D).

***Abdomen*.** Abdomen dark brown, gradually expanded to last tergite. Ultimate tergite broad, subquadrate; posteromedial extension rounded, with truncate posterior margin; weakly punctured; mostly hairy; median longitudinal sulcus present. Forceps dark brown, subcontiguous, symmetrical; bases strongly expanded, with two stout inner teeth; inner margin with giant tooth near apex and sparse denticles throughout; apex pointed, incurved. Penultimate sternite rounded, posterior margin weakly emarginate.

**Figure 9.**
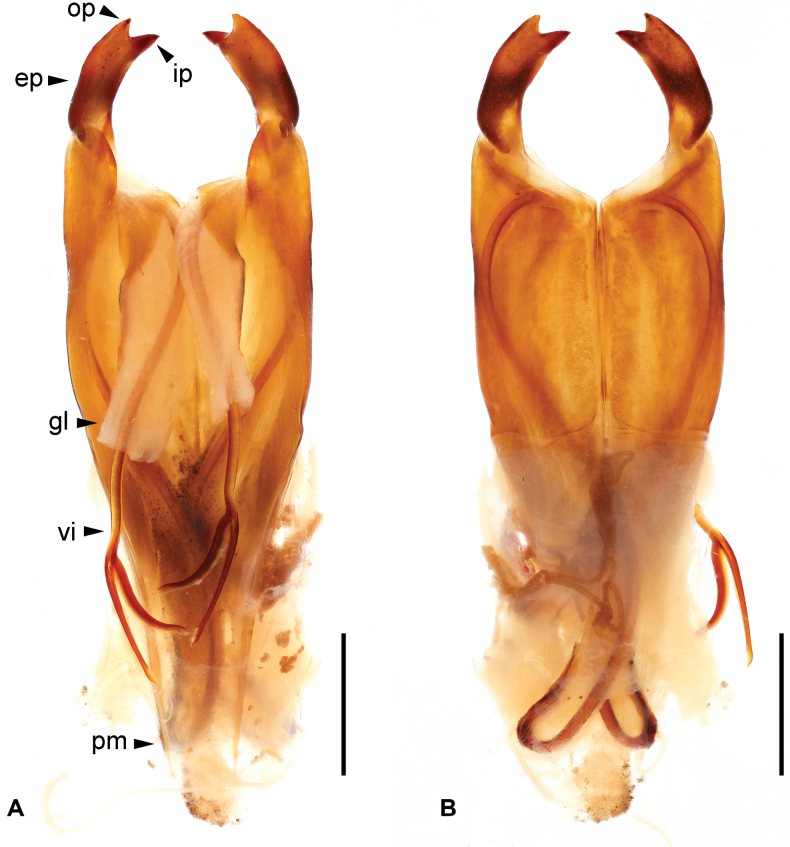
*Cranopygia longibifurcata* sp. nov., male genitalia. **A.** Dorsal view; **B.** Ventral view. Abbreviations: ep – external paramere; gl – genital lobe; ip – inner process; op – outer process; pm – paramere; vi – virga. Scale bars: 1 mm.

***Genitalia*.** Genitalia broad, brown (Fig. 9A, B). Paramere subtriangular basally. Genital lobes well developed; virga within genital lobe slender, apex bifurcated into a thick inner branch and a thin outer branch, each branch longer than external paramere, abruptly constricted apically. External paramere slender, with mostly consistent width; incision of anterior margin deep, wide; inner process subconical, pointed apically; outer process shorter than inner process, obtuse.

#### Etymology.

The specific epithet refers to the long bifurcate apex of the virga

#### Distribution.

The species is currently known only from Yunnan Province, China.

### ﻿ Cranopygia shidianensis sp. nov.

Taxon classificationAnimaliaDermapteraPygidicranidae

66728AA7-222F-54F8-83B4-37876C1FD6EC

https://zoobank.org/DA51C0F7-48C2-488E-A021-C190E2D52E1D

Figs 10, 11, 12

#### Specimens examined.

***Holotype***: China • ♂; Yunnan Province, Baoshan City, Shidian County, Guojiafenbaobao; 24.4611°N, 99.2853°E; 1210 m; 6.v.2025; Wei He leg. ***Paratype***: China •♀; same data as holotype. No additional non-type material examined.

#### Differential diagnosis.

This species belongs to the *marmoricrura*-group based on the genital lobe near half of the entire genitalia length, external paramere with a distinct outer process, and virga slender, slightly undulate, basally sclerotized, without lateral flanges, and unforked apically (Hincks 1959). Within this group, it most closely resembles *Cranopygia celebensis* (de Bormans, 1903) in male genital structure but differs in the shortened tegmina and concealed hindwings (vs. fully developed), penultimate sternite with an emarginate posterior margin (vs. rounded), and virga over three times as wide as that of *C. celebensis* (Hincks 1959; Steinmann 1986).

#### Description.

**Figure 10.**
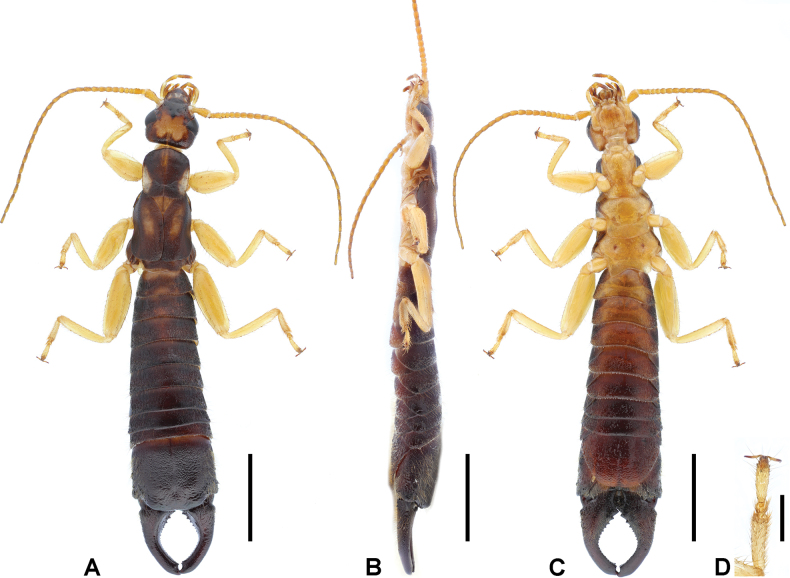
*Cranopygia shidianensis* sp. nov., male holotype. **A.** Habitus, dorsal view; **B.** Habitus, lateral view; **C.** Habitus, ventral view; **D.** Tarsomeres of left hind leg, dorsal view. Scale bars: 5 mm (**A–C**); 1 mm (**D**).

**Male. *General appearance*.** Median-sized, whole body mostly setose (Fig. 10A–C). Body length 28 mm. Forceps symmetrical, length 5 mm.

***Head*.** Head longer than broad; mostly dark brown, medially with one six-lobed pale area. Frontal and coronal sutures distinct. Eyes not prominent, about as long as genae. Antennae pale brown, with at least 29 segments; first antennal joint shorter than distance between antennal bases. Mouthparts brown to dark brown.

***Thorax*.** Pronotum slightly longer than wide; anterior and lateral margins rounded; posteromedial margin distinctly emarginate, forming two posterolateral lobes. Median longitudinal furrow distinct. Surface mostly dark brown, with pale anteromedian and lateral areas. Visible part of mesonotum pale. Tegmina shortened, near 1.5 times as long as pronotum; mostly dark brown, with a big pale area on anterior half of dorsal surface. Scales of hindwings strongly reduced, with only extreme end visible. Legs slender, pale; second tarsomere near as wide as third (Fig. 8D).

***Abdomen*.** Abdomen pale basally, other parts dark brown, gradually expanded to last tergite. Ultimate tergite broad, subquadrate; posteromedial extension rounded, with truncate posterior margin; weakly punctured; hairy laterally; median longitudinal sulcus present. Forceps dark brown, symmetrical, gently curved; bases strongly expanded; dorsal margin of inner surface with giant teeth basally and subapically, ventral margin fringed with small teeth and terminated by a giant subapical tooth; inner margin between subapical tooth and apex concaved. Penultimate sternite rounded, posterior margin emarginate.

**Figure 11.**
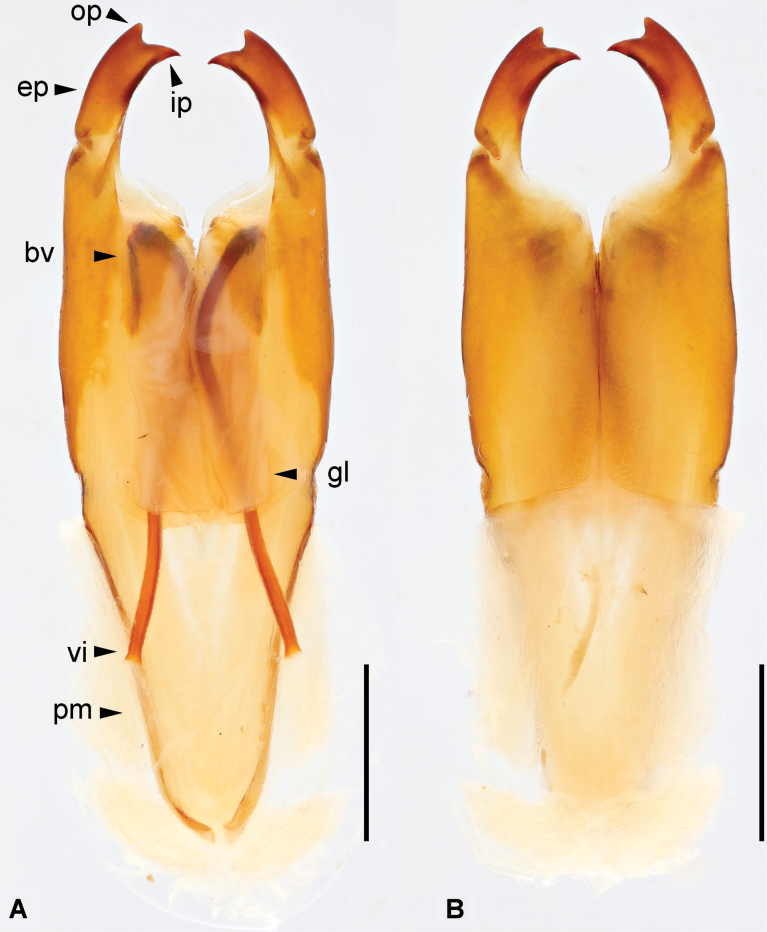
*Cranopygia shidianensis* sp. nov., male genitalia. **A.** Dorsal view; **B.** Ventral view. Abbreviations: bv – basal vesicle; ep – external paramere; gl – genital lobe; ip – inner process; op – outer process; pm – paramere; vi – virga. Scale bars: 1 mm.

***Genitalia*.** Genitalia slender, pale brown (Fig. 11A, B). Paramere subtriangular basally. Genital lobes well developed; virga within genital lobe slender, apex widened and truncate; basal vesicle sclerotized, short, thin. External paramere slender, with mostly consistent width; incision of anterior margin deep, wide; inner process subconical, pointed apically; outer process shorter than inner process, obtuse.

**Figure 12.**
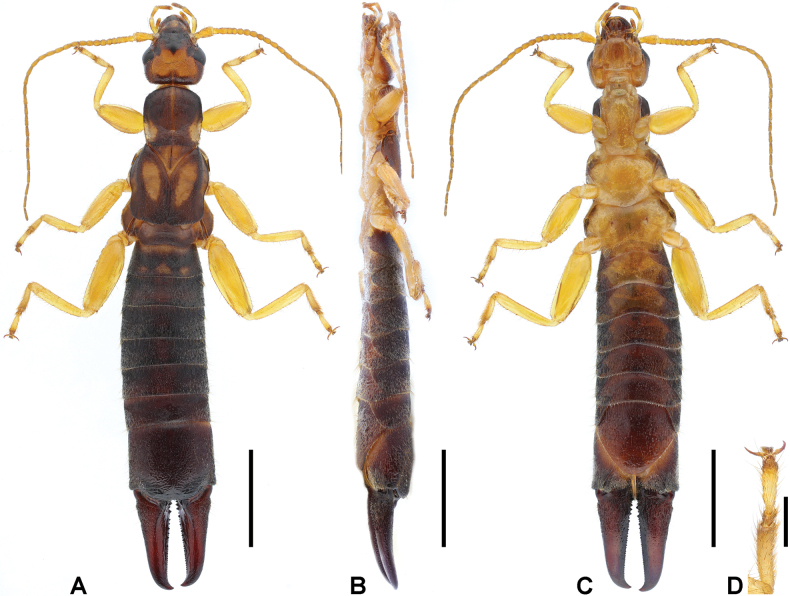
*Cranopygia shidianensis* sp. nov., female paratype. **A.** Habitus, dorsal view; **B.** Habitus, lateral view; **C.** Habitus, ventral view; **D.** Tarsomeres of left hind leg, dorsal view. Scale bars: 5 mm (**A–C**); 1 mm (**D**).

**Female.** Body shape and coloration similar to male (Fig. 12A–C). Body length 29.6 mm. Second tarsomere near as wide as third (Fig. 12D). Forceps length 5.4 mm. Forceps contiguous, tapering, symmetrical; apically incurved; inner margins mostly straight and denticulate, basal teeth larger than subsequent ones.

#### Etymology.

The new species is named after Shidian County, the type locality.

#### Distribution.

The species is currently known only from Yunnan Province, China.

### ﻿Key to Chinese species of *Cranopygia* based on males

**Table:** 

1	Virga simple, not forked apically, short (c. 1.5–2 × length of external paramere)	**2**
–	Virga forked apically; or if simple, then long (c. 3–5 × length of external paramere) with a well-developed, sclerotized basal vesicle	**5**
2	Forceps symmetrical, inner margins mostly diverging, with a large inner tooth near apex	***C. dravidia* (Burr, 1914)**
–	Forceps asymmetrical, tapering, inner margins subcontiguous, crenulate	**3**
3	Virga stout, strongly undulate, c. 2 × length of genital lobe	***C. modesta* (de Bormans, 1894)**
–	Virga slender, at most slightly undulate, c. 1.5 × length of genital lobe (*cumingi*-group)	**4**
4	Tegmina and hindwings well developed; inner process of external paramere with two equal apical teeth	***C. guizhouensis* Chen, 2024**
–	Tegmina shortened, hindwings absent; inner process of external paramere with one distinct apical tooth and one minute subapical tooth	***C. sauteri* (Burr, 1912)**
5	Virga simple, not forked apically	**6**
–	Virga forked apically (*appendiculata*-group)	**12**
6	External parameres with barely distinguishable outer process	***C. liuhuaishana* sp. nov.**
–	External parameres with well-developed outer process	**7**
7	Virga strongly curved, its junction with basal vesicle located at mid-length of paramere when not everted (*siamensis*-group)	**8**
–	Virga slender, straight or slightly undulate, its junction with basal vesicle located near anteromedial margin of paramere (*marmoricrura*-group)	**14**
8	Inner process of external paramere clearly bidentate apically	**9**
–	Inner process of external paramere unidentate	**11**
9	Virga simple, c. 3 × length of external paramere	***C. tonkinensis* Hincks, 1955**
–	Virga denticulate in apical one-third, c. 5 × length of external paramere	**10**
10	Pronotum wider than long, posteromedial margin weakly emarginate; inner process of external paramere with rounded apical incision; virga (in situ) outside of genital lobe nearly as long as genital lobe	***C. lisu* sp. nov.**
–	Pronotum longer than wide, posteromedial margin distinctly emarginate; inner process of the external paramere with subtriangular apical incision; virga (in situ) outside of genital lobe c. half length of genital lobe	***C. kongqueshana* sp. nov.**
11	Genital lobe c. 1.5 × length of external paramere; outer process of external paramere located at basal one-third; virga stout	***C. proxima* Hincks, 1959**
–	Genital lobe c. two-thirds length of external paramere; outer process of external paramere located at mid-length; virga slender	***C. siamensis* (Dohrn, 1863)**
12	Virga with one apical branch more than twice the length of the other	***C. appendiculata* Hincks, 1955**
–	Apical branches of virga subequal in length	**13**
13	Apical branches of virga slightly longer than external paramere; external paramere 3 × as long as wide	***C. longibifurcata* sp. nov.**
–	Apical branches of virga c. one-third length of external paramere; external paramere 2 × as long as wide	***C. yunnanea* Bey-Bienko, 1959**
14	Tegmina and hindwings well developed; lateral margins of external paramere diverging toward apex	***C. marmoricrura* (Audinet-Serville, 1839)**
–	Tegmina shortened, hindwings absent; lateral margins of external paramere nearly parallel	**15**
15	Inner and outer processes of external paramere similar in shape; virga c. 1.5 × length of genital lobe	***C. shidianensis* sp. nov.**
–	Inner process of external paramere longer and narrower than outer process; virga c. 2 × length of genital lobe	***C. vitticollis* (Stål, 1855)**

## ﻿Discussion

The discovery of five new *Cranopygia* species from South China expands the known diversity of the genus and highlights both taxonomic complexity and biogeographic interest within the group. These *Cranopygia* species are characterized by a second tarsomere that is nearly as wide as, or narrower than, the third tarsomere. This condition contrasts with that of *Tagalina* Dohrn, 1863, in which the second tarsomere is extraordinarily dilated, approximately three times as wide as the third (Hincks 1959). The new taxa fall into three established *Cranopygia* species groups (*siamensis*-, *picta*-, and *marmoricrura*-groups) and a tentatively delimited *appendiculata*-group, showing that the Chinese fauna encompasses a spectrum of morphological variation comparable to that recorded in Indo-Malayan regions.

Within Pygidicraninae, *Cranopygia* remains one of the most morphologically heterogeneous lineages. Male genital characters, particularly the shapes of parameres, the virga and its branches, and the genital lobes, continue to provide the most consistent, diagnostic features. At the same time, uneven or incomplete descriptions of historical types have fostered taxonomic uncertainty and the persistence of dubiously defined species (Steinmann 1986). Our results lend practical support to the species-group framework of Hincks (1959): each of the five taxa described here can be placed in an existing group or in a reasonable extension of one. However, some species groups may constitute convenience assemblages rather than natural clades (Kamimura et al. 2016); testing this will require broader sampling and molecular phylogenetic analysis.

The variation observed in wing development among the new species, ranging from fully developed tegmina and hindwings to strongly reduced or concealed wings, suggests potential evolution of flight reduction within *Cranopygia*. Such losses of flight capability are plausibly linked to habitat specialization: species with reduced wings are often associated with sheltered microhabitats (e.g., leaf litter, under bark or in karst crevices) where active dispersal is less important. The structural modifications of the virga and parameres observed in the new species may also indicate reproductive isolation mechanisms among sympatric species.

Biogeographically, the five species demonstrate that South China harbors a richer and more structurally diverse pygidicranine fauna than previously recognized. Yunnan and Guangxi, with their complex topography and mosaic of subtropical to tropical habitats, appear to function as both speciation centers and corridors linking Indochina and southern China. Future research should focus on the expanded sampling across South China and Indochina, the re-evaluation of species-group definitions using morphometric and molecular datasets, the exploration of ecological correlates of wing reduction and genital variation, and the conservation assessments of localized endemic species. These efforts will refine our understanding of *Cranopygia* evolution and provide a foundation for reconstructing the phylogeny of Pygidicranidae as a whole.

## Supplementary Material

XML Treatment for Cranopygia kongqueshana

XML Treatment for Cranopygia lisu

XML Treatment for Cranopygia liuhuaishana

XML Treatment for Cranopygia longibifurcata

XML Treatment for Cranopygia shidianensis

## ﻿Additional information

### Use of AI

No use of AI was reported.

### Author ORCIDs

Zhi-Teng Chen https://orcid.org/0000-0002-6331-8978

Chao Jiang https://orcid.org/0000-0003-1841-1169

### Data availability

All of the data that support the findings of this study are available in the main text.
